# Real-World Efficacy and Safety of a Topical Salvia fruticosa Leaf Extract Rich in Carnosic Acid and Carnosol for Mild to Very Severe Facial Seborrheic Dermatitis: A 12-Year Retrospective Study

**DOI:** 10.7759/cureus.113656

**Published:** 2026-07-30

**Authors:** Panagiotis Kallimanis, Serafim Prodromidis

**Affiliations:** 1 Pharmacy, Dr. Kallimanis' Community Pharmacy, Athens, GRC; 2 Dermatology, Private Dermatology Practice, Athens, GRC

**Keywords:** carnosic acid, carnosol, facial seborrheic dermatitis, greek sage, natural products, phytotherapy, retrospective case series, salvia fruticosa, seborrheic dermatitis, topical therapy

## Abstract

Objective

We aimed to evaluate the safety and efficacy of a cream (SDπ) containing *S. fruticosa* leaf extract rich in carnosic acid and carnosol in the treatment of facial seborrheic dermatitis (SD).

Methods

In this 12-year retrospective study, 10 patients (eight males, two females; age range, 40-78 years) with mild to very severe facial seborrheic dermatitis (mean baseline Seborrheic Dermatitis Area and Severity Index (SEDASI) score: 19; range, 10-60) applied SDπ cream once nightly as monotherapy. Follow-up ranged from 6 months to 5 years.

Results

All patients achieved complete disease clearance (SEDASI=0) within a mean of 7.7 days (range 3-15 days) with marked reduction in erythema, scaling, and rapid relief of pruritus. Remission persisted from 20 days to 10 months. Subsequent episodes responded to re-treatment in a shorter period (mean 4.8 days), while no relapses were observed during long-term maintenance therapy with nightly or alternate-night application. No local or systemic safety concerns were documented throughout treatment and the longitudinal follow-up period.

Conclusions

In this retrospective observational study, SDπ cream was associated with favorable clinical outcomes and good tolerability, suggesting that it may represent a promising natural topical monotherapy for facial seborrheic dermatitis. Although these findings are encouraging, prospective randomized controlled studies are required to confirm efficacy and long-term safety. To our knowledge, this work represents the first full-length retrospective report in the literature documenting the therapeutic role of *S. fruticosa* leaf extract rich in carnosic acid and carnosol in seborrheic dermatitis.

## Introduction

Seborrheic dermatitis (SD) is a chronic inflammatory skin disease of unclear etiology that predominantly affects areas rich in sebaceous glands, particularly on the face. The disease is clinically characterized by pruritus, erythema, and scaling and follows a lifelong course marked by alternating periods of exacerbation and remission. The global prevalence of SD in adults ranges from approximately 1% to 14.3%, with a higher incidence observed in males compared to females. Despite its high prevalence, the pathophysiological mechanisms underlying SD remain incompletely understood. Current evidence suggests a multifactorial origin involving *Malassezia *colonization, sebaceous gland activity, and individual host susceptibility factors, including immune and barrier dysfunctions. The chronic and recurrent nature of the disease has a substantial negative impact on patients’ quality of life [1,2].

Several drugs and treatments have been introduced to reduce skin lesions in seborrheic dermatitis. Topical corticosteroids and calcineurin inhibitors are commonly used to control inflammation in SD, whereas antifungal agents primarily exert their effects by reducing *Malassezia *proliferation [1,3]. Although these therapies are often effective, their use is limited by well-recognized adverse effects. Indeed, prolonged application of topical corticosteroids may result in skin atrophy, telangiectasia, and acneiform eruptions [4], while calcineurin inhibitors are frequently associated with transient burning, itching, and erythema [5]. In addition, the long-term use of certain antifungal agents, such as ciclopirox olamine and ketoconazole, remains controversial, as it may aggravate disease severity or contribute to reduced responsiveness over time [3]. Emerging evidence also indicates that some *Malassezia *species may exhibit decreased susceptibility to commonly used antifungal agents, further complicating long-term disease management [6]. Given these limitations, there is increasing interest in the development of novel topical therapies with a favorable safety profile.

*Salvia fruticosa *Mill. (Greek sage, syn. *Salvia triloba *L.) is a Mediterranean medicinal plant traditionally used for its anti-inflammatory, antimicrobial, and antioxidant properties. Its leaves are particularly rich in phenolic diterpenes, predominantly carnosic acid and its oxidative derivative carnosol, which are considered the main bioactive constituents [7]. Carnosic acid and carnosol have demonstrated potent anti-inflammatory activity via multiple signaling pathways, including NF-κB (nuclear factor kappa B), MAPK (mitogen-activated protein kinase), Nrf2 (nuclear factor erythroid 2-related factor 2), SIRT1 (sirtuin 1), STAT3 (signal transducer and activator of transcription 3), and NLRP3 (NOD-, LRR- and pyrin domain-containing protein 3) inflammasome [8].

The selection of *S. fruticosa *for the present study was based on both its unique phytochemical composition, characterized by a high content of the abietane diterpenes carnosic acid and carnosol, and the documented pharmacological activities of these compounds. Furthermore, the novelty of the present study lies in the clinical evaluation of a standardized *S. fruticosa *extract rich in carnosic acid and carnosol as monotherapy for facial seborrheic dermatitis, enabling the assessment of a well-defined phytochemical composition under real-world clinical conditions.

In our previous report, a standardized *S. fruticosa *extract rich in carnosic acid demonstrated promising preliminary clinical outcomes in plaque psoriasis [9]. Building upon these findings, the present study aimed to investigate its potential therapeutic role in seborrheic dermatitis.

The aim of this retrospective observational study was to evaluate the clinical outcomes, safety, and long-term follow-up of patients with facial seborrheic dermatitis treated with an oil-in-water cream (SDπ cream) containing a standardized *S. fruticosa *leaf extract rich in carnosic acid and carnosol.

We report the clinical outcomes of ten patients with mild to very severe facial SD treated exclusively with this formulation. To our knowledge, this is the first full-length clinical study evaluating a standardized *S. fruticosa *extract rich in carnosic acid and carnosol as monotherapy for facial seborrheic dermatitis. Preliminary findings from this work were previously presented in abstract form at the 73rd International Congress and Annual Meeting of the Society for Medicinal Plant and Natural Product Research (GA), Naples, Italy, 2025.

## Materials and methods

Plant material

*S. fruticosa *leaves were collected from Messinia, South Peloponnese, Greece, in the morning during the flowering period. The leaves were dried at room temperature in a well-ventilated area for 14 days.

Extract preparation

**Figure 1:**
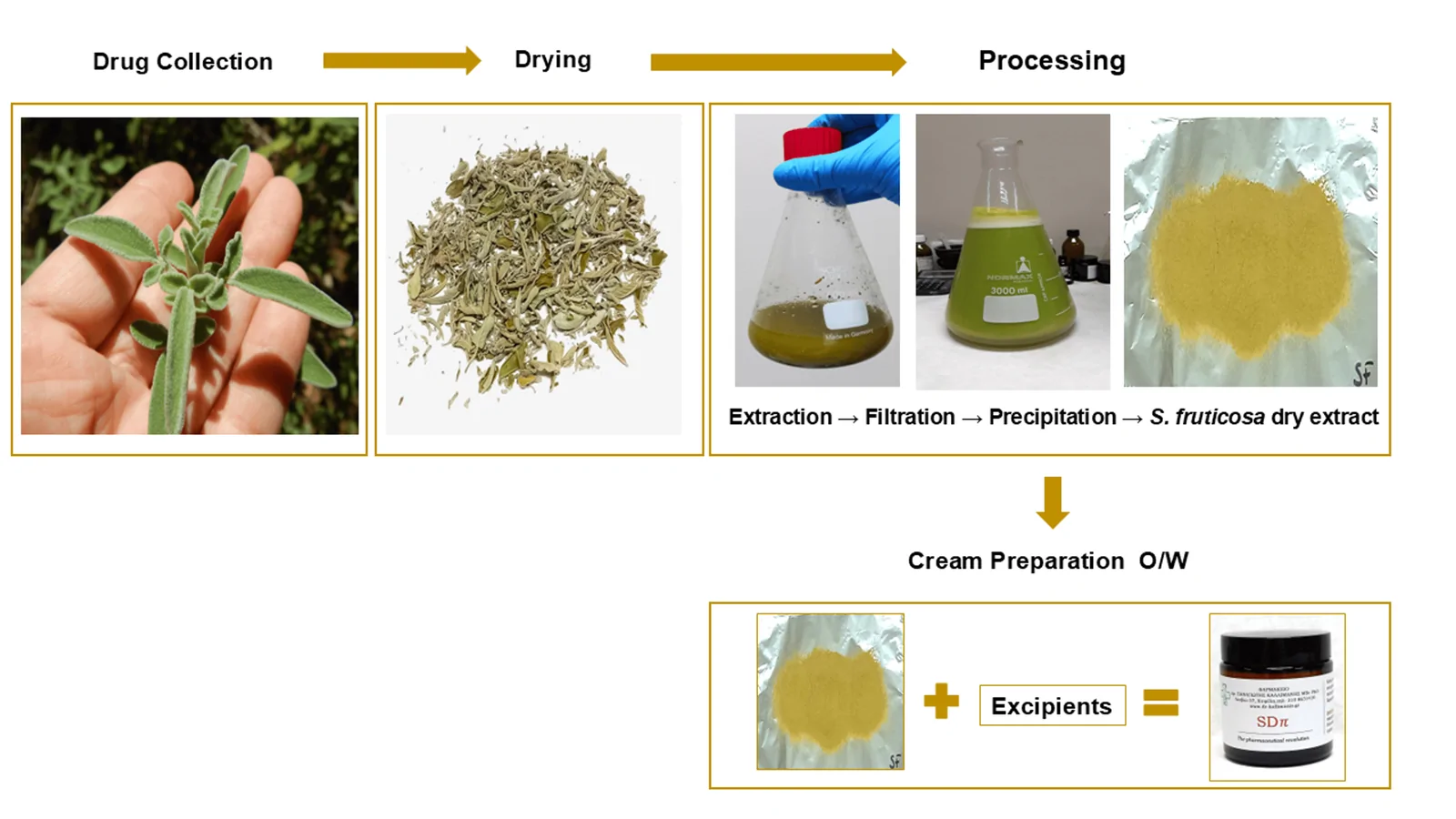
Preparation of Salvia fruticosa leaf extract and SDπ cream. *Salvia fruticosa *leaves were collected in Messinia, South Peloponnese, Greece, dried at room temperature, and processed to obtain a dry extract enriched in carnosic acid and carnosol. The extract was subsequently incorporated into an oil-in-water (O/W) cream with appropriate excipients to prepare SDπ cream. Source: All images in this figure are original photographs taken by the first author.

Briefly, the *S. fruticosa *leaf extract was prepared as follows: 100 g of dried *S. fruticosa *leaves, ground to a fine powder, were extracted with methanol at a 1:10 (w/v) ratio (100 g plant material in 1 L solvent) for 48 h at room temperature in an opaque container. Following extraction, the solid plant material was removed by filtration. The filtrate volume was subsequently adjusted to the initial volume (1 L) with methanol, after which 2 L of distilled water were added. The resulting precipitate was collected by filtration, dried, and finely powdered to obtain the corresponding *S. fruticosa *dry extract [10] (Fig. 1).

Extract chemical analysis

Qualitative and quantitative phytochemical characterization of the *S. fruticosa *extract was performed as previously described [10].

SDπ cream

The formulation was a rapidly absorbed, non-greasy oil-in-water cream containing a standardized *S. fruticosa *extract enriched in carnosic acid and carnosol (>15% combined content), along with pharmaceutically appropriate excipients [10]. The SDπ cream, as a galenic formulation, was prepared in the laboratory of Dr. Kallimanis' community pharmacy. It was formulated and prepared in accordance with Good Manufacturing Practice (GMP) guidelines [11] and the quality standards of the European Pharmacopoeia [12], using the same standardized manufacturing procedure for all batches. Because a patent protects the formulation, some manufacturing details cannot be disclosed.

Study design and patients

This retrospective observational study was based on data extracted from the clinical records of a private dermatology clinic and a community pharmacy. Patients were treated and followed between January 2012 and December 2024. The use of the *S. fruticosa-*based cream was initiated at the patients’ request, as they expressed a preference for a non-conventional, plant-derived therapeutic approach rather than standard pharmacologic treatments. A total of 10 patients (eight males and two females; age range, 40-78 years) with mild to very severe facial SD were enrolled in the study.

Diagnosis

The diagnosis was established clinically by the treating dermatologist.

Treatment

Patients applied SDπ cream once nightly to affected and surrounding skin as monotherapy.

Inclusion criteria

Patients with facial SD who had not received other treatment, topical or systemic drug, or cosmetic cream for their skin disease for at least 1 month before enrollment were included in the study.

Exclusion criteria

Patients with (1) a history of hypersensitivity to the active substances or any of the excipients, (2) pregnancy, or (3) lactation were excluded.

Disease severity

Disease severity was assessed using the Seborrheic Dermatitis Area and Severity Index (SEDASI), a validated scoring system for facial seborrheic dermatitis [11]. The index evaluates disease extent, lesion distribution, erythema, and scaling across four facial regions. Total scores range from 0 to 60 and are categorized as mild (1-14), moderate (15-29), severe (30-44), and very severe (≥45) [13]. SEDASI scores were assessed by the treating dermatologist.

Statistical analysis

Because of the descriptive nature of this retrospective observational study and the limited sample size, only descriptive statistics were performed. Continuous variables are presented as range, whereas categorical variables are presented as frequencies and percentages.

Patient satisfaction

Patient satisfaction was assessed using a single-item questionnaire scored on a 5-point Likert scale (1: very satisfied, 2: satisfied, 3: neither satisfied nor dissatisfied, 4: dissatisfied, 5: very dissatisfied).

Ethics statement

Human Subjects

Written informed consent was obtained from all patients for the publication of anonymized clinical data and clinical photographs. All data were anonymized prior to analysis. Clinical photographs were further anonymized by masking identifiable facial features before publication. The study was conducted in accordance with the principles of the Declaration of Helsinki.

## Results

A total of ten Caucasian patients with facial seborrheic dermatitis, with a higher prevalence of males (n = 8) compared to females (n = 2), were enrolled in the study. Most patients (6/10) had mild disease at baseline, while the remaining patients presented with moderate (2/10), severe (1/10), or very severe (1/10) disease according to the SEDASI classification. The mean baseline SEDASI score was 19 (range: 10-60). Treatment with the SDπ cream resulted in consistent clinical improvement across all cases. Complete clinical clearance was achieved in all patients, corresponding to a SEDASI score of 0, without concomitant therapy. The mean time to complete clearance was 7.7 days (range: 3-15 days). Clinical improvement was observed in all key disease parameters, including desquamation, erythema, and pruritus. Pruritus resolved early in the course of treatment, often within the first day of application. In some patients, a transient increase in desquamation was observed during the initial 2-3 days, followed by progressive resolution of scaling and erythema. Among patients who did not receive maintenance therapy, remission duration ranged from 20 days to 10 months (Table 1).

**Table 1:** Baseline demographic and clinical characteristics of patients with facial seborrheic dermatitis treated with SDπ cream. Data include patient demographics, disease duration, baseline disease severity according to the Seborrheic Dermatitis Area and Severity Index (SEDASI), treatment duration, follow-up period, duration of remission, and patient satisfaction score. *The severity of SD has been evaluated using SEDASI (Seborrheic Dermatitis Area and Severity Index). **Patient satisfaction - 1: very satisfied, 2: satisfied, 3: neither satisfied nor dissatisfied, 4: dissatisfied, 5: very dissatisfied. *** No additional relapses were observed after initiation of maintenance therapy. **** No relapse occurred while the patient remained on maintenance therapy.

No	Gender/Age	Disease duration (years)	SEDASI*	Clinical cure (days)	Follow-up (months)	Duration of remission	Patient satisfaction**
1	M/44	New-onset	60	7	60	10 months	1
2	M/64	25	10	15	12	20 days	1
3	M/40	24	20	7	36	25 days	1
4	M/53	1	20	10	12	20 days	1
5	M/56	6	10	8	8	45 days	1
6	M/43	26	10	5	12	90 days	1
7	F/76	4	10	3	24	30 days	1
8	F/64	New-onset	10	8	24	35 days***	1
9	M/ 78	New-onset	30	10	6	****	1
10	M/ 57	New-onset	10	4	12	25 days***	1

Relapses occurred in most patients during follow-up. Subsequent episodes were generally milder and responded more rapidly to re-treatment, requiring less time to achieve complete clearance compared with the initial treatment period (mean time to clearance: 4.8 days). Three patients received long-term maintenance therapy consisting of once-nightly or alternate-night application of the cream. One patient initiated maintenance therapy immediately after achieving complete clearance, whereas two initiated it following relapse. No relapses were observed during long-term maintenance therapy in any of these patients.

No local or systemic adverse events were observed during the study period, and long-term or maintenance use was well tolerated, including in patients with significant comorbidities and in a patient undergoing immune checkpoint inhibitor (nivolumab) therapy for malignancy.

Cosmetic tolerability and overall acceptability were rated as excellent by all participants. High levels of patient satisfaction were reported regarding the rapid onset of clinical improvement, easy application, once-nightly use, and the absence of an unpleasant or greasy residue. No evidence of treatment resistance was identified, even with prolonged or repeated use of the herbal formulation. Follow-up duration ranged from 6 months to 5 years, confirming the favorable safety profile and sustained clinical efficacy of the SDπ cream.

Patient 1

**Figure 2:**
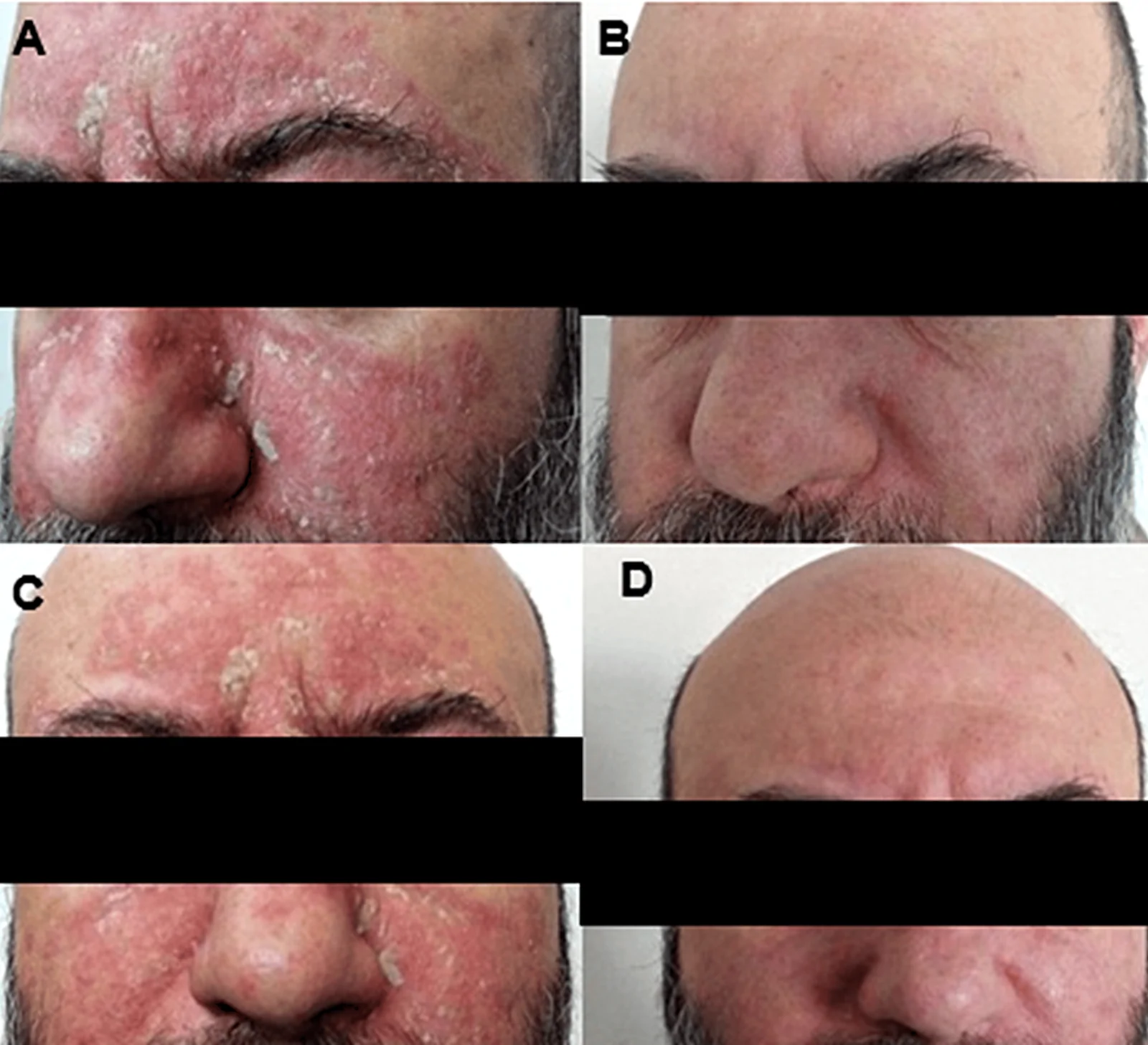
Clinical response of patient 1 with severe facial seborrheic dermatitis treated with SDπ cream. Clinical response of Patient 1 with severe facial seborrheic dermatitis (SEDASI = 60) treated with SDπ cream. Baseline images demonstrate extensive erythema and scaling involving the forehead, cheeks, and nasolabial areas. (A) Oblique view at baseline. (B) Oblique view after seven days of treatment. (C) Frontal view at baseline. (D) Frontal view after seven days of treatment, showing complete clinical clearance with resolution of erythema and scaling. SEDASI: Seborrheic Dermatitis Area and Severity Index

A 44-year-old male with liver cirrhosis presented with new-onset facial SD characterized by extensive erythema involving the forehead and bilateral nasal areas (SEDASI=60). Concomitant medications included propranolol, eplerenone, folic acid, and furosemide. The SDπ cream was applied once nightly to the entire face. Immediate relief of pruritus was reported, with transient increased desquamation during the first two days, followed by progressive resolution of erythema and scaling. Complete clinical clearance was achieved within seven days. Remission persisted for 10 months; a mild relapse responded fully to re-treatment within one week (Figure 2).

Patient 2

**Figure 3:**
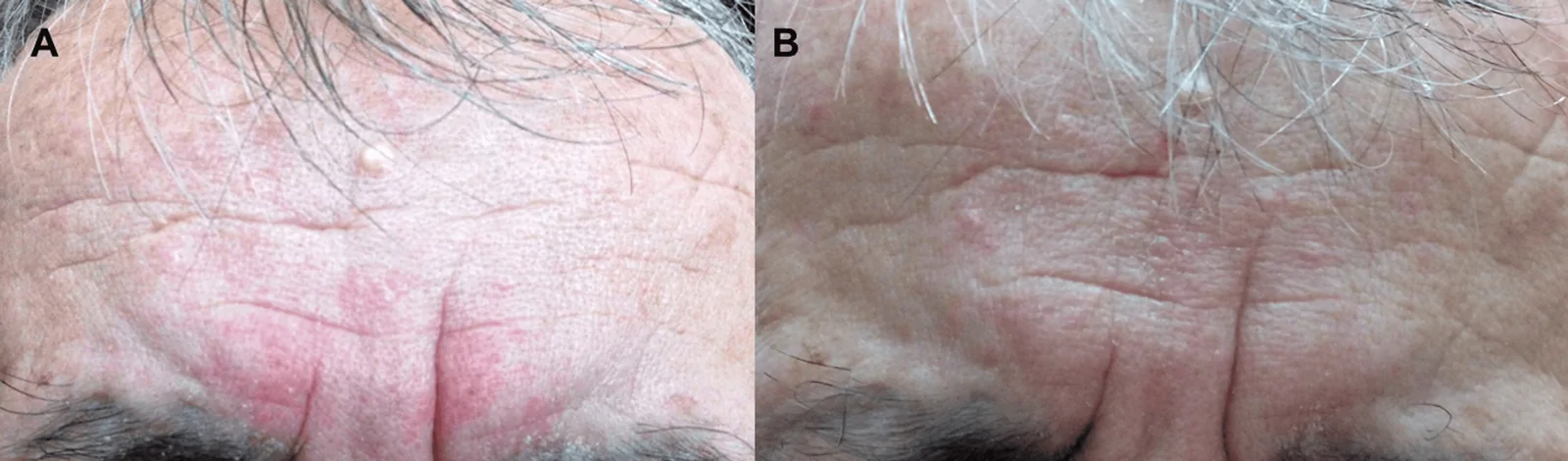
Clinical response of Patient 2 with facial seborrheic dermatitis and a 25-year disease history treated with SDπ cream. Clinical response of Patient 2 with long-standing facial seborrheic dermatitis (SEDASI = 10) treated with SDπ cream. (A) Baseline image demonstrating mild erythema of the forehead. (B) Complete clinical clearance after 15 days of once-nightly treatment with SDπ cream, with resolution of erythema. The patient had a 25-year history of seborrheic dermatitis and achieved complete remission despite inconsistent adherence to the treatment regimen. SEDASI: Seborrheic Dermatitis Area and Severity Index

A 64-year-old male with a 25-year history of SD presented with mild forehead erythema (SEDASI=10). The SDπ cream was applied once nightly. A transient increase in desquamation was observed during the first 2-3 days. The patient was receiving valsartan and amlodipine for hypertension and paroxetine for depression. Complete clinical clearance was achieved within 15 days, despite inconsistent adherence to the treatment regimen. After 20 days, mild signs of dermatitis were observed again. Re-treatment resulted in complete clearance within one week (Figure 3).

Patient 3

**Figure 4:**
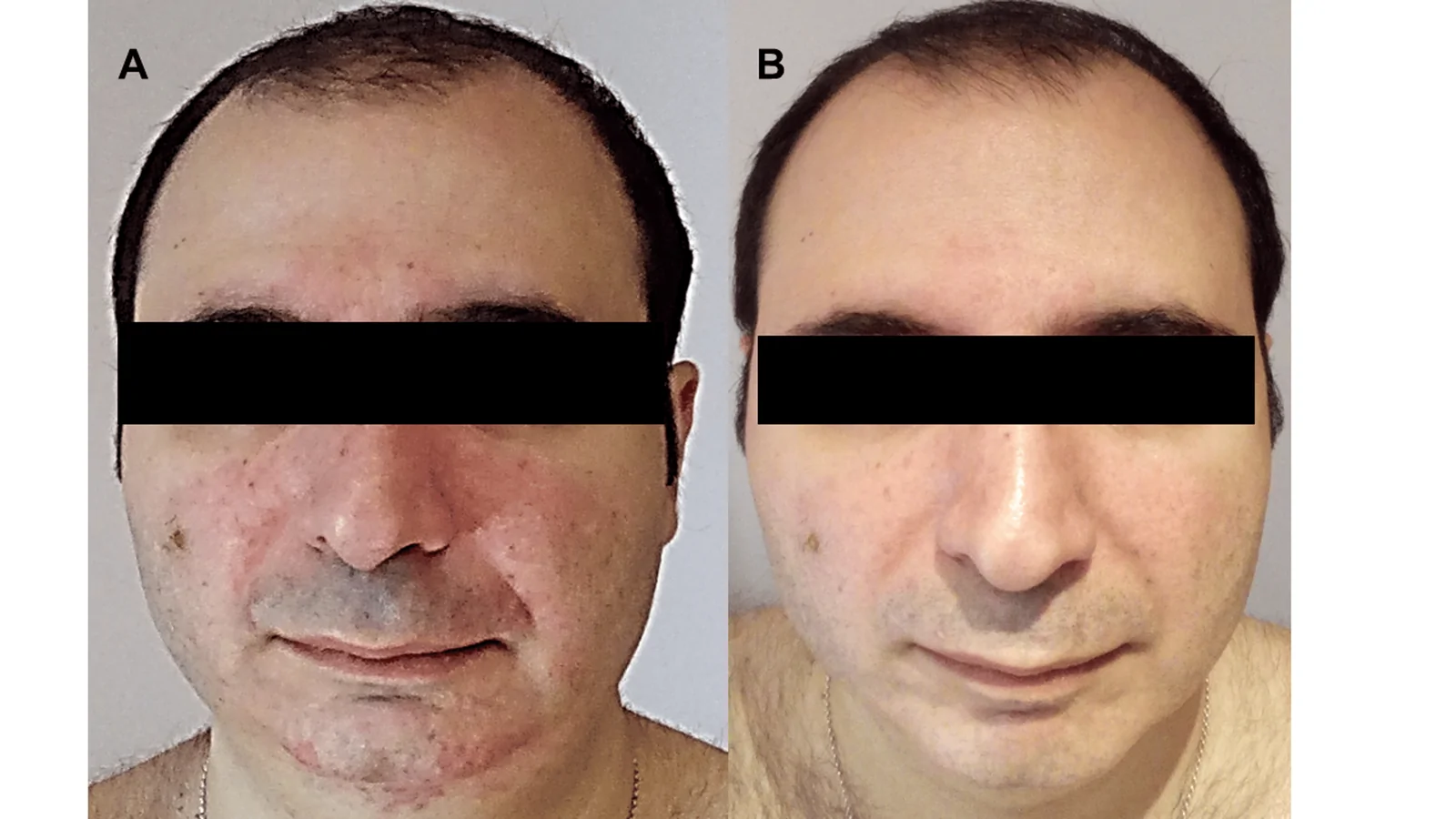
Clinical response of Patient 3 with recurrent facial seborrheic dermatitis treated with SDπ cream. Clinical response of Patient 3 with recurrent facial seborrheic dermatitis (SEDASI = 20) treated with SDπ cream. (A) Baseline image demonstrating erythema and scaling involving the cheeks, nasolabial areas, and chin. (B) Complete clinical clearance after seven days of treatment with SDπ cream, with resolution of erythema and desquamation. The patient remained relapse-free during three years of follow-up while receiving maintenance therapy. SEDASI: Seborrheic Dermatitis Area and Severity Index

A 40-year-old male with recurrent facial SD involving the forehead, bilateral nasal areas, and chin (SEDASI=20), initiated treatment with SDπ cream after discontinuing intermittent corticosteroid and antifungal therapy one month earlier. The cream was applied once nightly to the forehead and nasal areas and every morning to the chin after shaving. Complete clinical clearance was achieved within seven days. Mild relapse after one month resolved within 3-4 days following reapplication. Maintenance therapy (once-nightly or alternate-night application) was recommended. The patient remained relapse-free during 3 years of follow-up (Figure 4).

Patient 4

A 53-year-old male with a 1-year history of facial seborrheic dermatitis (SEDASI = 20) was treated with SDπ cream applied once nightly. Complete clinical clearance was achieved within 10 days. Mild relapse occurred after 20 days and responded completely to re-treatment.

Patient 5

A 56-year-old male with a 6-year history of facial seborrheic dermatitis (SEDASI = 10) applied SDπ cream once nightly. Complete clinical clearance was achieved within eight days. Remission lasted 45 days before a mild relapse, which resolved following re-treatment.

Patient 6

A 43-year-old male with a 26-year history of facial seborrheic dermatitis (SEDASI = 10) received once-nightly treatment with SDπ cream. Complete clinical clearance was achieved within five days. The patient remained relapse-free for 90 days during follow-up.

Patient 7

A 76-year-old female with a 4-year history of facial seborrheic dermatitis (SEDASI = 10) was treated with SDπ cream once nightly. Complete clinical clearance was achieved within three days. A mild relapse occurred after 30 days and responded completely to re-treatment.

Patient 8

A 64-year-old female with new-onset facial seborrheic dermatitis (SEDASI = 10) applied SDπ cream once nightly. Complete clinical clearance was achieved within eight days. Mild recurrence after 35 days responded completely to re-treatment, and no additional relapses occurred after initiation of maintenance therapy.

Patient 9

**Figure 5:**
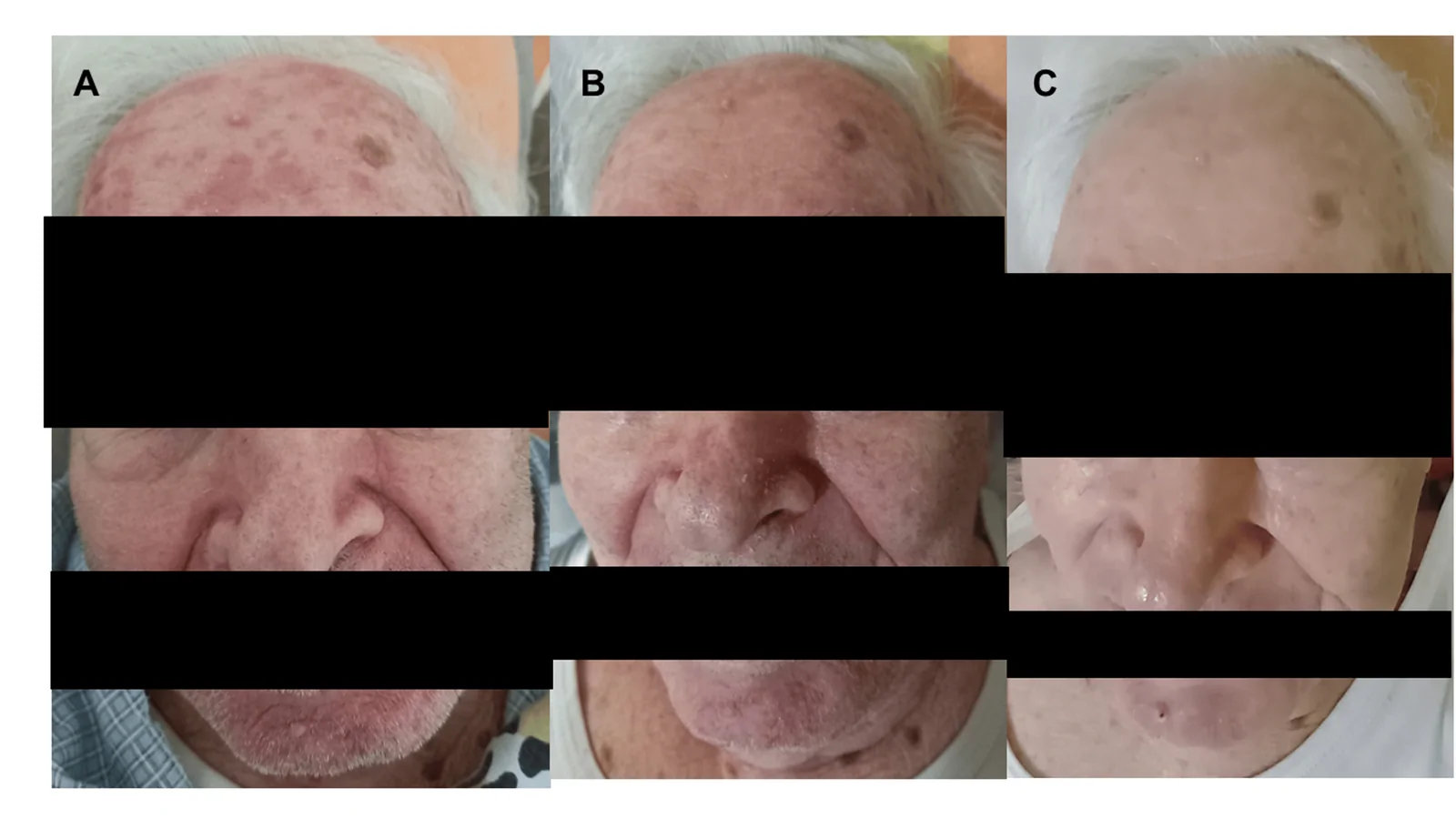
Clinical response of Patient 9 with refractory facial seborrheic dermatitis treated with SDπ cream. Clinical response of Patient 9 with extensive facial seborrheic dermatitis (SEDASI = 30) refractory to topical betamethasone valerate. (A) Baseline image demonstrating marked erythema and scaling of the forehead. (B) Substantial clinical improvement after six days of once-nightly treatment with SDπ cream. (C) Complete clinical clearance after ten days of treatment, with resolution of erythema and scaling. Continued nightly application prevented relapse. SEDASI: Seborrheic Dermatitis Area and Severity Index

A 78-year-old male with sudden-onset extensive facial seborrheic dermatitis (SEDASI=30), unresponsive to topical betamethasone valerate, initiated treatment with the SDπ cream applied once nightly. Marked improvement was noted by day six, with complete clearance by day ten. Continued nightly application prevented relapse (Figure 5).

Patient 10

**Figure 6:**
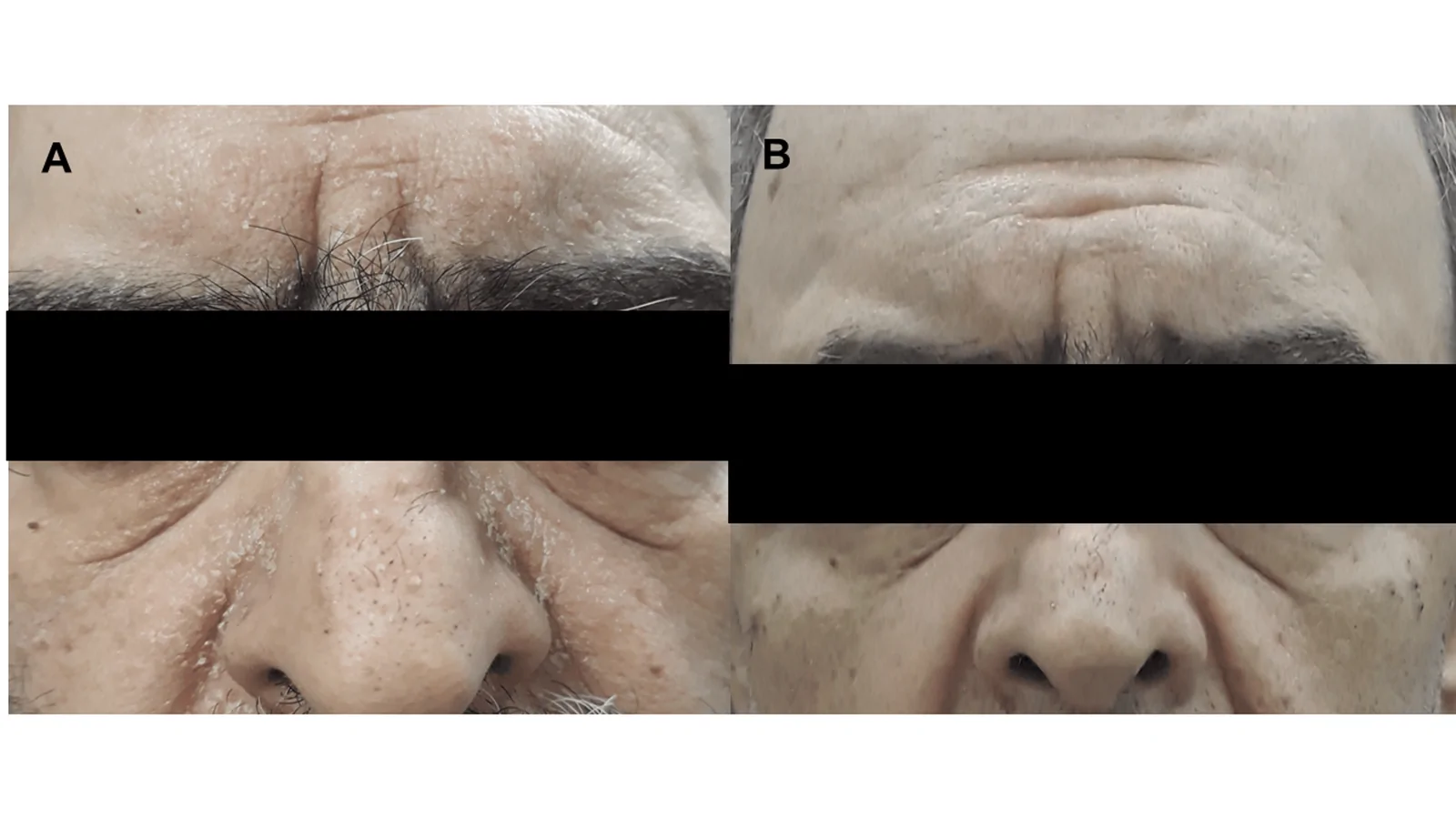
Clinical response of Patient 10 with nivolumab-associated facial seborrheic dermatitis treated with SDπ cream. Clinical response of patient 10 with nivolumab-associated facial seborrheic dermatitis (SEDASI = 10). (A) Baseline image demonstrating scaling involving the forehead and nasolabial areas in the absence of significant erythema. The patient reported intense pruritus and a burning sensation despite the relatively mild clinical appearance. (B) Complete clinical clearance after four days of once-nightly treatment with SDπ cream, with resolution of desquamation and symptoms. Recurrence during a subsequent nivolumab cycle responded completely to re-treatment, while maintenance therapy prevented further episodes during continued immunotherapy. SEDASI: Seborrheic Dermatitis Area and Severity Index

A 57-year-old male with laryngeal carcinoma developed facial seborrheic dermatitis following nivolumab immunotherapy (SEDASI=10). The SDπ cream, applied once nightly, resulted in complete clinical clearance within four days. Recurrence during a subsequent immunotherapy cycle resolved with re-treatment, and maintenance use prevented further episodes during subsequent nivolumab cycles (Figure 6).

Overall, treatment with SDπ cream demonstrated high efficacy as monotherapy for facial seborrheic dermatitis, providing rapid relief of pruritus and complete clinical clearance within a short treatment period in most patients. Clinical improvement was accompanied by prolonged remission during follow-up, while relapses responded promptly to re-treatment. Maintenance therapy effectively prevented further recurrences, and patient satisfaction was consistently high, with no treatment-related adverse effects reported throughout the study.

## Discussion

In this retrospective study, treatment with an oil-in-water cream (SDπ) containing *S. fruticosa *leaf extract rich in carnosic acid and carnosol resulted in complete clinical clearance of facial SD in all treated patients. All participants, with a mean baseline SEDASI score of 19, achieved a SEDASI score of 0 within a mean of 7.7 days, with marked improvement observed across all major clinical parameters, including erythema, scaling, and pruritus. Importantly, no local or systemic adverse events were reported during either the acute treatment phase or the long-term follow-up period (range: 6 months to 5 years).

The speed of clinical response observed in this series is noteworthy. Antifungal agents such as ketoconazole and ciclopirox typically require several weeks of treatment to achieve meaningful clinical improvement, while topical corticosteroids, although often effective in the short term, are limited by the risk of cutaneous adverse effects and rebound flares upon discontinuation [1,3-4]. In contrast, the SDπ cream was associated with prompt symptom resolution, with pruritus frequently subsiding within the first days of application, suggesting anti-inflammatory and antipruritic activity.

An additional clinically relevant finding was the durability of remission and the favorable response to re-treatment. Relapses occurred in most patients during follow-up; however, subsequent episodes were generally milder and resolved more rapidly than the initial episode, with a mean clearance time of 4.8 days. Three patients received long-term maintenance therapy consisting of nightly or alternate-night application of the cream. Notably, all remained relapse-free, irrespective of whether maintenance therapy was initiated immediately after disease clearance or following relapse. This observation is particularly relevant given the chronic and relapsing nature of SD and the need for safe long-term management strategies.

Unpublished clinical observations with a carnosic acid-rich *Salvia rosmarinus *(rosemary) leaf extract provide additional context regarding tolerability patterns. While similar clinical responses were observed in preliminary clinical observations, subtle differences in early tolerability emerged. Specifically, a transient flare during the first 1-2 days of treatment was consistently reported with the rosemary extract, a reaction not observed with the *S. fruticosa *preparation. In addition, clinical clearance appeared to be achieved 1-2 days earlier with *S. fruticosa*. Although these observations are preliminary and were not derived from a formal comparative study, they may reflect differences in the phytochemical composition of the two extracts despite their partially overlapping profiles.

Weckesser et al. evaluated a *Salvia officinalis *extract (carbon dioxide/isopropyl alcohol, 14-20:1 w/w) containing carnosic acid and carnosol (35% w/w diterpene phenols) against *Malassezia furfur *using the agar dilution method and reported non-evaluable antifungal activity [14]. Therefore, the favorable clinical outcomes observed in the present study are unlikely to be explained by direct antifungal effects against *Malassezia*. Rather, these effects may be attributed to the multimodal biological activity of *S. fruticosa *and its major phenolic diterpenes, carnosic acid and carnosol. Indeed, both compounds exert potent anti-inflammatory activity through modulation of multiple signaling pathways, including NF-κB, MAPK, STAT3, and NLRP3 inflammasomes, which are implicated in the inflammatory component of seborrheic dermatitis [8,15-16]. In addition, their strong antioxidant properties may mitigate oxidative stress in the epidermis, a factor increasingly recognized as contributing to barrier dysfunction and inflammation in SD [16]. Moreover, carnosic acid and carnosol have been identified as activators of peroxisome proliferator-activated receptor gamma (PPARγ) [17]. Activation of this receptor has been shown to stimulate keratinocyte differentiation and promote epidermal homeostasis [18]. Furthermore, the pleiotropic effects of PPARγ include maintenance of skin barrier integrity, modulation of immunoinflammatory signaling, normalization of cutaneous lipid metabolism, regulation of the cutaneous microbiome, and modulation of neuroimmune pathways [19]. Although these observations derive primarily from studies in atopic dermatitis [19], several of these biological processes are also implicated in the pathogenesis of seborrheic dermatitis [16], further supporting a possible contribution of PPARγ activation to the therapeutic effects observed in the present study. This multimodal profile may also explain the absence of treatment resistance, even with prolonged or repeated use.

These findings suggest a favorable safety profile in populations where the use of topical corticosteroids or systemic agents may be problematic. Cosmetic acceptability and patient satisfaction were uniformly high, an important consideration for chronic facial dermatoses where adherence is strongly influenced by formulation characteristics. Notably, the SDπ cream was well tolerated in elderly patients and in individuals with significant comorbidities, including liver cirrhosis and a malignancy treated with immune checkpoint inhibitor therapy.

A multifactorial condition such as SD may benefit from equally multifaceted therapeutic approaches. Natural products, due to their pleiotropic activity, may offer a rational therapeutic strategy by targeting multiple pathogenic pathways simultaneously while maintaining a favorable tolerability profile.

Several limitations of this study should be acknowledged. First, its retrospective observational design precludes the establishment of causal relationships. Second, the relatively small sample size and inclusion of patients from a single private dermatology practice may limit the generalizability of the findings. Although consecutive eligible patients were included according to predefined inclusion and exclusion criteria, the inclusion of patients who opted for a plant-derived treatment approach may have introduced some degree of selection bias. Furthermore, clinical diagnosis and outcome assessment were performed by the treating dermatologist without independent blinded evaluation, introducing the possibility of observer bias. Moreover, patient satisfaction was assessed retrospectively using a simple ordinal scale and may have been influenced by desirability bias, particularly because responses were obtained within the treating clinical setting. In addition, the absence of a control group and randomization precludes direct comparison with established topical therapies. Nevertheless, the study also has several strengths, including the long-term real-world follow-up, the use of a standardized treatment protocol with the formulation administered as monotherapy, assessment with a validated disease severity index (SEDASI), and the consistency of clinical responses across patients with varying disease severity, long disease duration, and prior treatment exposure. To our knowledge, this is the first full-length retrospective report investigating the clinical efficacy of *S. fruticosa *in seborrheic dermatitis.

## Conclusions

This retrospective study suggests that topical treatment with *S. fruticosa *leaf extract rich in carnosic acid and carnosol was associated with favorable clinical outcomes and may represent a promising monotherapy for mild to very severe facial seborrheic dermatitis. Complete disease clearance was achieved in all treated patients. When relapses occurred, they responded to re-treatment, while maintenance therapy was associated with sustained remission and prevention of subsequent relapses.

Given the chronic and relapsing nature of seborrheic dermatitis, these findings support the potential clinical relevance of this natural topical therapeutic approach. Although the results are encouraging, they should be interpreted in light of the retrospective study design, limited sample size, and absence of a control group, which preclude definitive conclusions regarding efficacy. Prospective, randomized, double-blind, controlled trials are required to confirm the efficacy, safety, and long-term outcomes of this therapeutic approach.

## References

[1] Dessinioti C, Katsambas A (2013). Seborrheic dermatitis: etiology, risk factors, and treatments: facts and controversies. Clin Dermatol.

[2] Turchin I, Albrecht L, Hanna S (2025). Current understanding of seborrheic dermatitis: epidemiology, burden of disease, and pathophysiology. J Cutan Med Surg.

[3] Dall'Oglio F, Nasca MR, Gerbino C, Micali G (2022). An overview of the diagnosis and management of seborrheic dermatitis. Clin Cosmet Investig Dermatol.

[4] Coondoo A, Phiske M, Verma S, Lahiri K (2014). Side-effects of topical steroids: a long overdue revisit. Indian Dermatol Online J.

[5] Safarini OA, Keshavamurthy C, Patel P (2026 Jan-). Calcineurin inhibitors. StatPearls [Internet].

[6] Nakamura Y, Kano R, Murai T, Watanabe S, Hasegawa A (2000). Susceptibility testing of Malassezia species using the urea broth microdilution method. Antimicrob Agents Chemother.

[7] Kallimanis P (2022). Study of plants of the Lamiaceae family for the production of metabolites that inhibit the action of the aryl hydrocarbon receptor (AhR) with application to skin diseases [PhD Thesis]. National and Kapodistrian University of Athens, Athens, Greece.

[8] Habtemariam S (2023). Anti-inflammatory therapeutic mechanisms of natural products: insight from rosemary diterpenes, carnosic acid and carnosol. Biomedicines.

[9] Kallimanis P, Prodromidis S (2026). Treating mild to severe plaque psoriasis topically with Salvia fruticosa Mill. rich in carnosic acid: an observational retrospective study. Planta Med.

[10] Kallimanis P (2026). Espacenet - Bibliographic data - Rosemary and sage extracts for skin diseases: compositions and treatment methods - Greek Patent GR1009073. https://worldwide.espacenet.com/publicationDetails/biblio?II=0&ND=3&adjacent=true&locale=en_EP&FT=D&date=20170707&CC=GR&NR=1009073B&KC=B.

[11] European Commission (2023). EudraLex. Volume 4. The Rules Governing Medicinal Products in the European Union: Good Manufacturing Practice (GMP) Guidelines. https://health.ec.europa.eu/medicinal-products/eudralex/eudralex-volume-4_en.

[12] Council of Europe (2022). European Pharmacopoeia. https://pheur.edqm.eu/.

[13] Micali G, Lacarrubba F, Dall'Oglio F, Tedeschi A, Dirschka T (2017). A new proposed severity score for seborrheic dermatitis of the face: SEborrheic Dermatitis Area and Severity Index (SEDASI). J Am Acad Dermatol.

[14] Weckesser S, Engel K, Simon-Haarhaus B, Wittmer A, Pelz K, Schempp CM (2007). Screening of plant extracts for antimicrobial activity against bacteria and yeasts with dermatological relevance. Phytomedicine.

[15] Park HR, Oh JH, Lee YJ (2021). Inflammasome-mediated Inflammation by Malassezia in human keratinocytes: a comparative analysis with different strains. Mycoses.

[16] Khimuk S, Andrusiewicz A, Mijas D, Nowicka D (2026). Molecular mechanisms in seborrheic dermatitis-systematic review. Int J Mol Sci.

[17] Rau O, Wurglics M, Paulke A (2006). Carnosic acid and carnosol, phenolic diterpene compounds of the labiate herbs rosemary and sage, are activators of the human peroxisome proliferator-activated receptor gamma. Planta Med.

[18] Mao-Qiang M, Fowler AJ, Schmuth M (2004). Peroxisome-proliferator-activated receptor (PPAR)-gamma activation stimulates keratinocyte differentiation. J Invest Dermatol.

[19] Bao Y, Xu K, Du Y (2026). PPARγ: a key orchestrator of epidermal barrier, immune responses, and lipid metabolism in atopic dermatitis pathogenesis and therapy. Front Allergy.

